# Visual Hand Recognition in Hand Laterality and Self-Other Discrimination Tasks: Relationships to Autistic Traits and Positive Body Image

**DOI:** 10.3389/fpsyg.2020.587080

**Published:** 2020-12-03

**Authors:** Mayumi Kuroki, Takao Fukui

**Affiliations:** Graduate School of Systems Design, Tokyo Metropolitan University, Hino, Japan

**Keywords:** visual hand recognition, hand laterality, self-other discrimination, autistic traits, body image

## Abstract

In a study concerning visual body part recognition, a “self-advantage” effect, whereby self-related body stimuli are processed faster and more accurately than other-related body stimuli, was revealed, and the emergence of this effect is assumed to be tightly linked to implicit motor simulation, which is activated when performing a hand laterality judgment task in which hand ownership is not explicitly required. Here, we ran two visual hand recognition tasks, namely, a hand laterality judgment task and a self-other discrimination task, to investigate (i) whether the self-advantage emerged even if implicit motor imagery was assumed to be working less efficiently and (ii) how individual traits [such as autistic traits and the extent of positive self-body image, as assessed *via* the Autism Spectrum Quotient (AQ) and the Body Appreciation Scale-2 (BAS-2), respectively] modulate performance in these hand recognition tasks. Participants were presented with hand images in two orientations [i.e., upright (egocentric) and upside-down (allocentric)] and asked to judge whether it was a left or right hand (an implicit hand laterality judgment task). They were also asked to determine whether it was their own, or another person’s hand (an explicit self-other discrimination task). Data collected from men and women were analyzed separately. The self-advantage effect in the hand laterality judgment task was not revealed, suggesting that only two orientation conditions are not enough to trigger this motor simulation. Furthermore, the men’s group showed a significant positive correlation between AQ scores and reaction times (RTs) in the laterality judgment task, while the women’s group showed a significant negative correlation between AQ scores and differences in RTs and a significant positive correlation between BAS-2 scores and dprime in the self-other discrimination task. These results suggest that men and women differentially adopt specific strategies and/or execution processes for implicit and explicit hand recognition tasks.

## Introduction

Over time, hands have developed in a remarkably human-specific manner and serve as an important interface between human’s external worlds (including other people) and their individual selves. In the past two decades, neuroimaging studies have revealed that a specific area of the brain [the extrastriate body area (EBA)] is selectively activated when perceiving body parts (e.g., Downing et al., 2001; Peelen and Downing, 2005; see Peelen and Downing, 2007 for a review). It is known that the right EBA responds more to allocentric view of body parts than to egocentric view and this preference in the right EBA for allocentric view is associated with social cognition (e.g., self-other discrimination; Chan et al., 2004; Saxe et al., 2006). Furthermore, Bracci et al. (2010) found a hand-preferring region in the EBA, suggesting that representations of the hand in the extrastriate visual cortex are distinct from representations of other body parts.

Many studies have investigated mental rotation tasks using hand stimuli (i.e., hand laterality judgment tasks) since the pioneering works in the field of cognitive psychology (Cooper and Shepard, 1975; Sekiyama, 1982; Parsons, 1987). These hand laterality judgment tasks developed from an object mental rotation task (Shepard and Metzler, 1971). The judgment task requires participants to determine whether a visual hand stimulus (which could be presented in several angular orientations) is a right or left hand. Reaction times (RTs) are linearly modulated by angular orientations of stimuli when performing a classic object (or letter) mental rotation task, which suggests that participants are mentally rotating the stimuli toward the familiar (i.e., the upward) position. However, the RTs in a hand laterality judgment task are not linearly modulated by the angular orientations of hand stimuli. Indeed, RTs depend on biomechanical constraints. Specifically, longer RTs were observed in this task, as it requires the biomechanically difficult mental rotation of a hand’s image, in comparison with a task that is biomechanically easier, even when the necessary stimulus rotation is equal (Parsons, 1994; Parsons et al., 1998; de Lange et al., 2006). Furthermore, the hand posture adopted by participants during this task also influences their RTs (Ionta et al., 2007; Ionta and Blanke, 2009). Specifically, longer RTs for hand laterality judgments were shown when participants were holding both hands behind their back than when the same task was performed with both hands placed on their knees. The biomechanical effect is stronger when the hands were presented from palm than from back, because physically rotating palms is assumed to be more difficult than rotating backs of hands (Sekiyama, 1982; Parsons, 1987, 1994; Gentilucci et al., 1998; ter Horst et al., 2010; Bläsing et al., 2013; Zapparoli et al., 2014; Conson et al., 2020). These results indicate that hand laterality judgment tasks involve implicit motor imagery processes. However, some studies did not reveal the effect of biomechanical constraints on the RTs (e.g., Lust et al., 2006; Steenbergen et al., 2007). ter Horst et al. (2010) suggested that engagement in motor imagery when performing a hand laterality judgment task depends on the used number of axes of rotation of the stimulus set.

In the last decade, research has increasingly addressed how people distinguish between their own hands and the hands of others (e.g., Aranda et al., 2010; Rossetti et al., 2010). Frassinetti and colleagues (Frassinetti et al., 2008, 2009) found a “self-advantage” effect, whereby self-related body stimuli are processed faster and more accurately than other-related body stimuli. Ferri et al. (2011), however, reported that the self-advantage effect emerged only when performing a hand laterality judgment task in which the participant did not explicitly need to recognize the identity of the hand (i.e., an implicit hand recognition task), and noted that this effect did not emerge in an explicit self-recognition task (i.e., a self-other discrimination task). Conson et al. (2015) also ran these visual hand recognition tasks (hand laterality judgment and self-other discrimination tasks), replicating the results of Ferri et al. (2011), and suggested that implicit motor imagery processes are essential for the emergence of the self-advantage effect (see also Frassinetti et al., 2011). Furthermore, concerning self-other discrimination tasks, Conson et al. (2015) found a “self-disadvantage” effect that led to longer RTs for the self-hand image, when compared with RTs for other-hand images (see also Ferri et al., 2011).

Recently, several studies using these visual hand recognition tasks have investigated task performance in clinical populations in comparison with healthy participants. In Campione et al. (2017), women outpatients diagnosed with eating disorders [*N* = 15; mean (± SD) age = 18.0 ± 2.1; age range = 15–21] were recruited, and they did not show the self-advantage effect in a hand laterality judgment task (although the RTs of these outpatients were comparable to those of the control participants). Furthermore, Conson et al. (2016) found that individuals with autism spectrum disorder [ASD; 18 participants (1 woman); mean (± SD) age = 14.6 ± 4.2; age range = 10–20] showed significantly longer RTs in comparison with typically developing (TD) peers in a classical hand laterality judgment task in which self-hand images were not used (see also Conson et al., 2013).

These studies investigating clinical populations inspired a research question concerning whether and how the extent of a person’s autistic traits and body appreciation affect their performance of implicit and explicit hand recognition tasks, compared to the same tasks performed by members of a population of TD participants. It is known that ASD is more common in males than females, with a male-to-female ratio of about 4 to 1 across the whole autism spectrum (Baird et al., 2006), and rising to 8 or 9 to 1 in higher-functioning samples (Mandy et al., 2011). By contrast, eating disorders are predominantly found in females, with a female-to-male ratio from about 4 to 1 during adolescence and about 10 to 1 in adulthood (Striegel-Moore and Bulik, 2007; Reijonen et al., 2016). Previous studies (e.g., Linn and Petersen, 1985; Voyer et al., 1995; Collins and Kimura, 1997) demonstrated that women exhibit inferior visuospatial performance, compared to men. The task involved in the hand laterality judgment calls for deciding whether the presented hand image is a right or left hand. Left-right discrimination is essential in everyday life, but many people report difficulties discriminating left from right in daily life, which results in left-right confusion. Sex differences in this confusion based on self-reported data have been found, indicating that women are more prone to left-right confusion than men are (e.g., Hannay et al., 1990).

With the above mind, we wondered whether and how sex difference affects the performance of implicit and explicit hand recognition tasks, even in those populations in which people have not been diagnosed. Indeed, Mochizuki et al. (2019) and Conson et al. (2020) recently investigated the effect of sex differences on the performance of a classical hand laterality judgment task, but the effect of sex differences on the performance of implicit and explicit hand recognition tasks (using self‐ and other-hand images) remains an open question. Answering this question will contribute to our further understanding of self-other discrimination and body part recognition.

During previous visual hand recognition tasks, the numbers of orientation conditions for the hand image were manipulated. For example, the numbers Ferri et al. (2011) and Conson et al. (2015) used were four and six, respectively (see also Ferri et al., 2012; Conson et al., 2017; De Bellis et al., 2017). It is assumed that the task would be easy and that implicit motor imagery processes would work less efficiently if the number of orientation conditions of the hand image is limited to two (i.e., upright and upside-down). Therefore, the current study aims to investigate whether the self-advantage effect emerges in the hand-laterality task and whether the self-disadvantage effect is revealed in the self-other discrimination task, even when the number of orientation conditions of the hand image in each visual hand recognition task is limited to two [egocentric (upright; 0°) and allocentric (upside-down; 180°); cf. Chan et al., 2004; Saxe et al., 2006; Conson et al., 2010].

The present study investigated how the individual traits of TD university students (who had not been diagnosed) modulated their performance in hand laterality judgment and self-other discrimination tasks using the Autism Spectrum Quotient (AQ) test (Baron-Cohen et al., 2001; Japanese version: Wakabayashi et al., 2004) and the Body Appreciation Scale-2 (BAS-2; Tylka and Wood-Barcalow, 2015; Japanese version: Namatame et al., 2017). Specifically, we focused on whether and how the differences in parameters (e.g., accuracy, reaction time, etc.) between self and other conditions, as an index of self-(dis)advantage, are modulated according to the scores of individual traits (the AQ and the BAS-2) in implicit and explicit hand recognition tasks.

## Materials and Methods

### Participants

In total, 36 right-handed university students [20 men, mean age (± SD): 21.4 ± 1.4] participated in the following visual hand recognition tasks. Of these participants, six were excluded because of their outlier performances (see Results section for details), leaving 30 participants (15 men) for inclusion in the analysis. All participants were confirmed to be right-handed [the minimum score among the participants was 30, and with a mean score (± SD) of 88.0 ± 16.3], as assessed by the Edinburgh Handedness Inventory (Oldfield, 1971). They had normal or corrected-to-normal vision, and none had any motor or sensory abnormalities. This study was approved by the ethics committee of Tokyo Metropolitan University’s Hino Campus, and all participants provided written informed consent according to the Declaration of Helsinki. They were naive to the purpose of the experiment and were paid for their participation.

### Apparatus

#### Photographing Participants’ Hands

A digital camera (PowerShot SX620 HS, Canon, Tokyo, Japan) installed in a box on a table (see Procedure section for details) was used, and stimuli were produced using Adobe Photoshop 2019.

#### Visual Hand Recognition Tasks (Laterality Judgment and Self-Other Discrimination Tasks)

**Figure 1:**
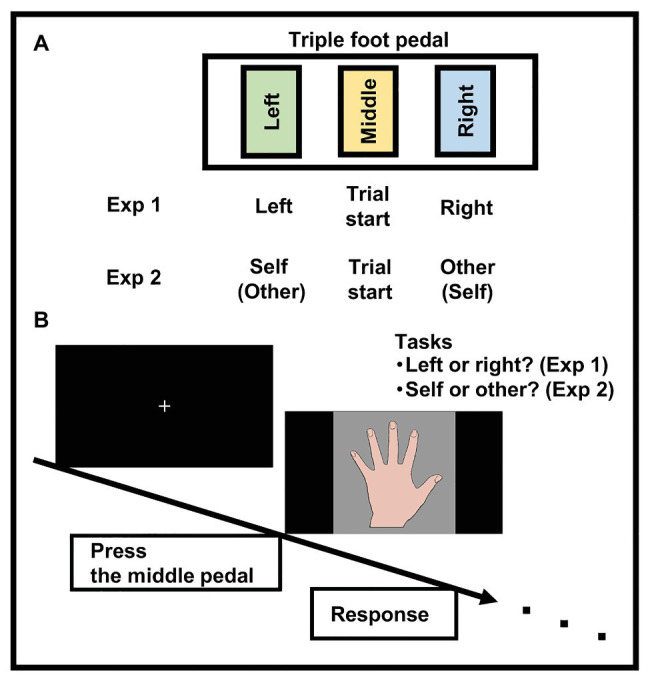
Experimental setup. Button assignment of triple foot pedal for response. Middle button was assigned for the trial start **(A)**. A time sequence of the trial. Participants pressed the middle pedal after a white cross appeared on the screen, and then a hand image (in the experiment, a full-color photograph was used) appeared, prompting a response **(B)**.

A laptop PC (ZenBook Pro UX550VD-7700. AsusTek Computer Inc., Taipei, Taiwan; 15.6 inches, screen resolution = 1,920 × 1,080 pixels) was used to present the stimuli and for data acquisition. Reacting with the hand itself could influence the performance in a visual hand recognition task, so participants were required to perform the task using their preferred foot with a triple foot pedal (RI-FP3BK. Route-R Corporation, Tokyo, Japan, see Figure 1A).

#### Questionnaire

The Autism Spectrum Quotient test (Baron-Cohen et al., 2001; Japanese version: Wakabayashi et al., 2004) is one of the most well-known questionnaires for measuring autistic traits. It consists of five subscales (i.e., social skill, attention switching, attention to detail, communication, and imagination). Each subscale has 10 items, resulting in a 50-item questionnaire (i.e., the maximum possible score is 50). If the score is 33 (the cutoff score in the Japanese version) or higher, that participant could possibly have a clinically significant level of autistic traits.

The Body Appreciation Scale-2 (Tylka and Wood-Barcalow, 2015; Japanese version: Namatame et al., 2017) is a widely used 10-item questionnaire on positive body image. This is a revision of the original 13-item BAS (Avalos et al., 2005). The average score of the 10 items is the participant’s final BAS-2 score. The maximum possible score is 5, with higher scores indicating higher levels of body appreciation.

### Procedure

#### Photographing Participants’ Hands

Participants sat comfortably on a chair and placed their hands in a box on a table. Their palms were touching the surface of the table, and the backs of the hands were photographed using a box on the ceiling of which a rail was installed for sliding the digital camera to take pictures of the right and left hands easily. Participants could not see the inside the box, and they were asked, prior to participating, to agree to have their hands photographed.

Full-color photos were made of the participants’ left and right hands (i.e., backs of the hands) against a gray background (1,063 × 1,063 pixels, see Figure 1B). One hand image was located in the center of each stimulus. The original images of the hands (one picture per hand) in the upright orientation (0°, consistent with the perspective of the viewer, i.e., upright) were digitally manipulated to obtain the hand images at an opposite orientation (180°, i.e., upside-down). These two orientations of the hand images (0 and 180°) were used as stimuli.

#### Visual Hand Recognition Tasks

About 1 week after their hands were photographed, participants engaged in two visual hand recognition tasks (i.e., a laterality judgment task and a self-other discrimination task). All participants performed the laterality judgment task first, followed by the self-other discrimination task on the same day (cf. Campione et al., 2017). We adopted this order to minimize participants’ attention to hand ownership when completing the laterality judgment task.

##### Laterality Judgment Task

Participants viewed the display on a laptop computer from a distance of approximately 60 cm while seated comfortably on a chair in front of the table on which the laptop was located. A triple foot pedal was situated beneath the participant’s feet, and the center of the pedal was aligned with the participant’s sagittal plane. Each participant was required to remove their shoes and to react to the stimuli using their right or left foot (Figure 1A). Concerning the response foot when starting the trial (i.e., pressing the middle button, Figure 1A), half of the participants began each trial with the right foot, while the other half started with the left foot. Once underway, the foot used could not be changed during the experiment. Participants were also required to place their hands in their lap, and their hands were concealed by a towel.

At the beginning of the trial, a white cross (i.e., a fixation point, 1.7 × 1.7°) was presented in the center of the display. When participants intended to start the trial, they pressed the middle pedal of the triple foot pedal. Immediately after pressing the pedal, a hand stimulus (18.4 × 18.4°) was presented in the center of the display. Participants were instructed to decide whether the presented hand stimulus was an image of a right or left hand and to respond as quickly and accurately as possible (Figure 1B). The right pedal was assigned for responding to the right hand images, and the left pedal was assigned for responding to the left hand images. For the other hand images, the hands of two sex-matched persons whom the participants did not know (i.e., the authors’ colleagues and other participants) were used.

The experiment consisted of 180 trials [= 2 (hand image laterality) × 2 (visual perspective conditions) × 3 (person’s hands, i.e., own and two same-sex others) × 15 trials (in each)], and participants took a 2-min break every 60 trials. Before the test trials, each participant performed 16 practice trials to ensure that they were performing the trials according to the instructions.

##### Self-Other Discrimination Task

The experimental procedure for the self-other discrimination task was the same as the one used in the laterality judgment task, except that the participants’ reactions to the right or left hand image were replaced by a reaction to the participants’ own hands or the hands of another person of the same sex.

The right pedal was assigned for responding to the self-hand images, while the left pedal was assigned for responding to the other person’s hand images for half of the participants. The pedal assignment for responding was reversed for the other half of the participants.

#### Questionnaire

All participants completed the Japanese pencil-and-paper versions of the AQ (Wakabayashi et al., 2004) and BAS-2 (Namatame et al., 2017) tests after performing the visual hand recognition tasks. The items in the AQ (e.g., “It does not upset me when my daily routine is disturbed”) are answered on a 4-point Likert scale (“definitely agree,” “slightly agree,” “slightly disagree,” and “definitely disagree”), while the participants rated each item of the BAS-2 (e.g., “Despite its flaws, I accept my body for what it is”) to indicate whether the question was true about the participant’s approval and acceptance of their own body, noted on a 5-point scale (i.e., never = 1, seldom = 2, sometimes = 3, often = 4, and always = 5).

### Data Processing and Analysis

#### Visual Hand Recognition Tasks

Accuracy and RTs were recorded in each condition. For each participant, RT outliers were removed by excluding trials with RTs that fell more than three SDs from the median of all trials. ANOVAs were conducted on accuracy and RTs, with sex (women and men) as a between-participants factor, and with hand ownership (self and other), hand image laterality (left and right), and visual perspective (upright and upside-down) as within-participant factors.

The sensitivity and response criterion for visual hand recognition were calculated as dprime and ln(*β*), respectively, according to the signal detection theory. The calculation of these two values was based on the formula reported by Macmillan and Creelman (1991). These values were also entered into ANOVAs, with sex (women and men) as a between-participants factor, and with two within-participant factors, namely, hand ownership (self and other) and visual perspective (upright and upside-down) in the laterality judgment task and hand laterality (left and right) and visual perspective (upright and upside-down) in the self-other discrimination task. Bonferroni-corrected *post-hoc* comparisons were performed when necessary.

#### Relationships Between Task Performance and Questionnaires’ Scores

Two questionnaire scores (i.e., AQ and BAS-2) were calculated for each participant. To determine whether and how individual traits would be related to ability in visual hand recognition, non-parametric Kendall rank correlation coefficients between each questionnaire score (AQ and BAS-2) and each parameter of task performance [accuracy, RTs, dprime, ln(*β*) (these values were averaging all conditions), and the differences in accuracy and RTs between the self and other conditions] were computed by sex. When computing each correlation coefficient, two levels (i.e., men and women) were set in the experimental design, so *α* = 0.05/2 = 0.025 was considered to be statistically significant, using the Bonferroni correction.

## Results

Data from the six participants whose accuracy or RTs did not fall within the range of the mean values ± two SDs in each visual recognition task were excluded from the data analysis. Therefore, the data of 30 participants (15 men) were included in the following results.

### Accuracy

Overall accuracy was high in both tasks at 96.2% in the laterality judgment task and 98.2% in the self-other discrimination task (see Table 1).

**Table 1:** Mean accuracy % (standard deviation) in the laterality judgment (top) and self-other discrimination (bottom) tasks (0°: upright, 180°: upside-down).

	Self				Other			
	Left		Right		Left		Right	
	0°	180°	0°	180°	0°	180°	0°	180°
**Laterality judgment task**								
Men	98.7	96.0	96.0	92.4	98.0	96.2	96.0	92.4
	(2.8)	(4.9)	(5.5)	(9.0)	(3.0)	(6.4)	(6.2)	(7.6)
Women	98.7	97.8	97.3	96.9	97.3	94.9	98.0	93.1
	(3.7)	(4.1)	(5.5)	(5.0)	(4.4)	(6.0)	(3.7)	(7.8)
**Self-other discrimination task**								
Men	96.0	98.2	98.2	99.1	99.1	98.9	99.3	99.3
	(8.7)	(3.1)	(4.0)	(2.3)	(2.0)	(2.4)	(1.4)	(1.9)
Women	96.4	99.1	98.7	96.9	99.1	98.4	98.0	96.4
	(4.3)	(3.4)	(3.7)	(7.1)	(2.0)	(3.5)	(3.3)	(1.9)

#### Laterality Judgment Task

The main effect of laterality was significant [*F*(1, 28) = 7.941, *p* = 0.009, partial *η*^2^ = 0.221; left hand image: 97.2 ± 4.6%; right hand image: 95.3 ± 6.7%]. The significant main effect of visual perspective [*F*(1, 28) = 6.297, *p* = 0.018, partial *η*^2^ = 0.184] was also noted. Furthermore, a second-order interaction among sex, ownership, and visual perspective (sex × ownership × visual perspective interaction) was noted [*F*(1, 28) = 4.411, *p* = 0.045, partial *η*^2^ = 0.136], and a *post-hoc* comparison revealed that accuracy concerning the self-image (97.3%) was higher than that concerning the other image (94.0%) on the upside-down image condition when the participant group was women, but no such significant difference was noted when the participant group was composed of men.

#### Self-Other Discrimination Task

No significant main effects on factors and no interactions were noted (*p* > 0.071).

### Reaction Times

#### Laterality Judgment Task

**Figure 2:**
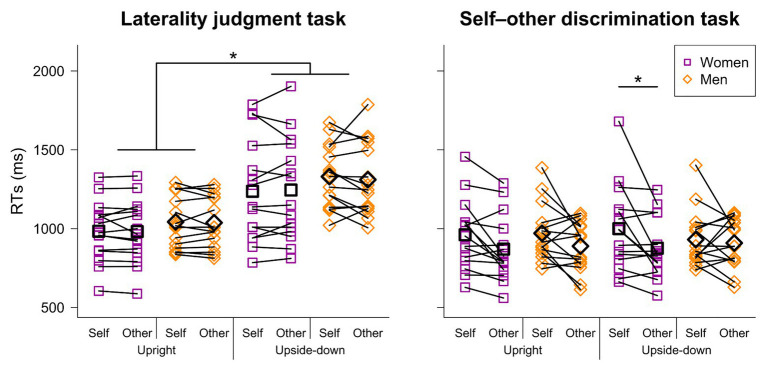
Reaction times (RTs) in the laterality judgment and self-other discrimination tasks. While no significant difference between the self and other conditions was noted in the laterality judgment task, RTs for the other hand image were significantly shorter than those for the self-hand image when the upside-down image was presented in the self-other discrimination task (see the text for detailed results). A black-lined square and diamond in each condition indicate mean values for women and men, respectively.

A significant main effect of visual perspective [*F*(1, 28) = 53.792, *p* < 0.001, partial *η*^2^ = 0.658] and a significant interaction between hand image laterality and visual perspective [*F*(1, 28) = 4.809, *p* = 0.037, partial *η*^2^ = 0.147] were noted (Figure 2). The results indicate that RTs for the upright image were shorter than those for the upside-down image, regardless of the hand laterality (1,037 vs. 1,273 ms, when a left hand image was presented; 985 vs. 1,288 ms, when a right hand image was presented). It is noteworthy that shorter RTs for the self-hand image, which were observed in previous studies (e.g., Ferri et al., 2011), were not revealed in the present experiment.

#### Self-Other Discrimination Task

We found a significant main effect of ownership [*F*(1, 28) = 7.557, *p* = 0.010, partial *η*^2^ = 0.213], a significant interaction between sex and visual perspective [*F*(1, 28) = 4.292, *p* = 0.048, partial *η*^2^ = 0.133] and a significant second-order interaction among sex, ownership, and visual perspective [*F*(1, 28) = 7.491, *p* = 0.011, partial *η*^2^ = 0.211]. A *post-hoc* comparison revealed that RTs for the other hand image (876 ms) were shorter than those for the self-hand image (1,000 ms) on the upside-down image condition when the participant group was women, but no such significant difference was noted when the participant group was composed of men (Figure 2).

### Dprime

#### Laterality Judgment Task

We found significant main effects of ownership [*F*(1, 28) = 10.798, *p* = 0.003, partial *η*^2^ = 0.278], visual perspective [*F*(1, 28) = 6.774, *p* = 0.015, partial *η*^2^ = 0.195], and a significant interaction between ownership and visual perspective [*F*(1, 28) = 4.475, *p* = 0.043, partial *η*^2^ = 0.138]. A *post-hoc* comparison revealed that dprime for the other hand image (3.82) was greater than that for the self-hand image (3.43) when an upright hand image was presented.

#### Self-Other Discrimination Task

A significant interaction between sex and hand laterality was noted [*F*(1, 28) = 6.164, *p* = 0.019, partial *η*^2^ = 0.180], but a *post-hoc* comparison revealed that no significant differences were found.

### In(*β*)

#### Laterality Judgment Task

No significant main effects on factors and no interactions were noted (*p* > 0.087).

#### Self-Other Discrimination Task

No significant main effects on factors and no interactions were noted (*p* > 0.122).

### Questionnaires

Participants’ AQ scores ranged from 6 to 34, with a mean AQ score of 19.4 (SD = 6.3), and no significant difference between the men’s and women’s groups was noted [*t*(28) = 0.369, *p* = 0.715]. The BAS-2 scores ranged from 1.5 to 4.3, with a mean BAS-2 score of 2.9 (SD = 0.8), and no significant difference between the men’s and women’s groups was noted [*t*(28) = −0.824, *p* = 0.417].

No significant correlation between AQ scores and BAS-2 was found in either the men’s group (*p* = 0.430) or the women’s group (*p* = 0.409).

### Correlation Coefficients Involved With AQ Scores

#### Laterality Judgment Task

**Figure 3:**
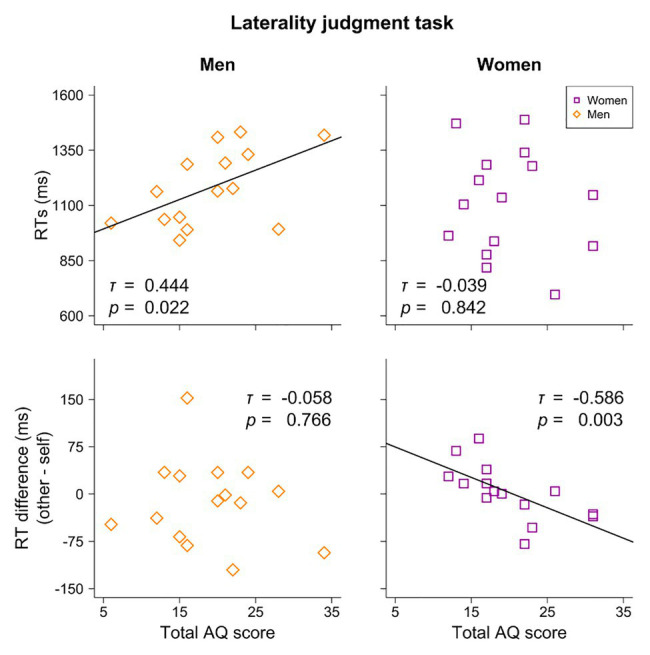
Correlations between Autism Spectrum Quotient (AQ) scores and RTs and between AQ scores and the difference in RTs (other – self) in the laterality judgment task. A significant positive correlation between AQ scores and RTs was noted in the men’s group, while a significant negative correlation between AQ scores and the difference in RTs was noted in the women’s group.

A significant correlation between total AQ scores and RTs was found in the men’s group (*τ* = 0.444, *p* = 0.022), indicating an increase in RTs in tandem with higher AQ scores (Figure 3, top left).

As for the women’s group, the difference in RTs between the self and other conditions (i.e., RTs in the other condition – RTs in the self condition) correlated significantly and negatively with total AQ scores (*τ* = −0.586, *p* = 0.003; Figure 3, bottom right). This result indicates that the lower AQ participants showed a quicker response to the self-image, in comparison with the other image, while the response pattern of the (relatively) higher AQ participants to the self-image was similar to that of the other image, or was reversed (i.e., there was a quicker response to the other image).

#### Self-Other Discrimination Task

No significant correlations between total AQ scores and each parameter of task performance were found in either the men’s or the women’s groups (*p* > 0.206).

### Correlation Coefficients Involved With BAS-2 Scores

#### Laterality Judgment Task

Total BAS-2 scores were not significantly correlated with any parameters of task performance in either the men’s or the women’s groups (*p* > 0.110).

#### Self-Other Discrimination Task

In the men’s group, the total BAS-2 scores were not significantly correlated with any parameters of task performance (*p* > 0.273).

**Figure 4:**
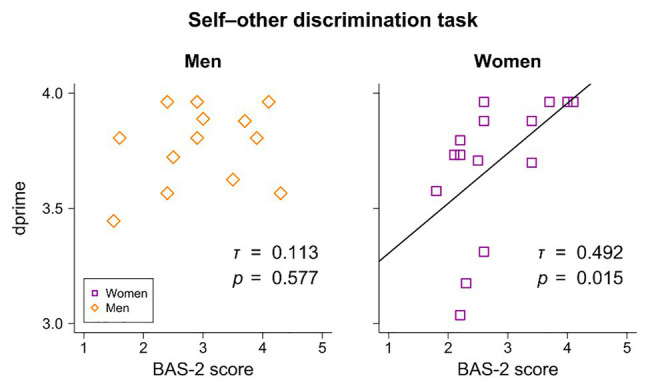
Correlations between Body Appreciation Scale-2 (BAS-2) scores and dprime in the self-other discrimination task. A significant positive correlation between BAS-2 scores and dprime was noted only in the women’s group.

In the women’s group, the correlation between BAS-2 and dprime was also significant (*τ* = 0.492, *p* = 0.015), indicating an increase in dprime according to higher BAS-2 scores (Figure 4).

## Discussion

The present study explored whether and how the extent of participants’ autistic traits and body appreciation affect the performance of implicit and explicit hand recognition tasks in a TD population, divided by sex (cf. Mochizuki et al., 2019; Conson et al., 2020). In addition, we investigated whether the results of visual recognition tasks (i.e., laterality judgment and self-other discrimination tasks) in previous studies (e.g., Ferri et al., 2011; Conson et al., 2015) would be replicated, even if the number of orientation conditions of the hand image was limited to two (i.e., upright and upside-down).

Concerning the self-advantage in the laterality judgment task, previous studies (Ferri et al., 2011; Frassinetti et al., 2011; Conson et al., 2015) suggested that this task requires motor simulation based on implicit sensorimotor knowledge about the body’s parts (in this case, the hands), including a combined visuo-sensorimotor strategy (cf. Brady et al., 2011; Ní Choisdealbha et al., 2011). The present task, in which the orientation had just two conditions (i.e., 0 and 180°), did not show this self-advantage. This result suggests that having only two orientation conditions is not enough to trigger this motor simulation (cf. ter Horst et al., 2010). This is consistent with the argument made by Conson et al. (2015), that implicit self-advantage is enhanced by a high “sensorimotor load.”

Concerning the results of the self-other discrimination (explicit) task, we found shorter RTs in the other condition than in the self-condition, findings that are in agreement with previous studies (e.g., Ferri et al., 2011; Conson et al., 2015). Conson et al. (2010) performed a highly similar task in a sample of right-handed men and showed an interaction among hand ownership, hand image laterality, and visual perspective, such as significantly faster RTs in recognizing others’ left hands than in recognizing others’ right hands, from an allocentric perspective (for right-handed male participants). Our results did not replicate those of Conson et al. (2010), which showed a tight association between self-other discrimination and egocentric/allocentric views. Regarding the women’s group, we found significantly shorter RTs for the other hand image (876 ms), compared to the self-hand image (999 ms) when the upside-down image was presented (i.e., the image was presented in an allocentric manner), resulting from the lengthened RTs for the upside-down self-image. The explicit task requires a mere visual representation of one’s own body (Frassinetti et al., 2011; Conson et al., 2015); therefore, this result was caused by women’s inferior performance in visual mental rotation (Linn and Petersen, 1985; Voyer et al., 1995; Collins and Kimura, 1997), in addition to visual unfamiliarity with self-image from an allocentric view. Concerning the women’s strategy in the hand laterality judgment task, Conson et al. (2020) recently investigated these using images of the backs and palms of hands. It is said that hand images viewed from the back often induce a visual strategy, while images viewed from the palm induce a motor strategy in the classical hand laterality task (Sekiyama, 1982; Parsons, 1987, 1994; Gentilucci et al., 1998; ter Horst et al., 2010; Bläsing et al., 2013; Zapparoli et al., 2014; Conson et al., 2020). Based on this assumption, Conson et al. (2020) argued that women would best represent an image viewed from the back as a self-hand and mainly adopt this visual strategy, while men best represented an image viewed from the palm as their own hand and mainly adopted the motor strategy.

We further investigated how individual traits (such as autistic traits and the extent of positive body image) would relate to performance in the laterality judgment (implicit) and self-other discrimination (explicit) task in the men’s and women’s groups. A significant relationship to the AQ score was revealed only in the laterality judgment task (not in the self-other discrimination task). Specifically, longer RTs aligned with a higher AQ score were shown in the men’s group but not in the women’s group. Although, to our knowledge, there have been no previous studies investigating the variation in RTs according to AQ scores in the laterality judgment task, some studies investigating the task performance of individuals with ASD, in comparison with TD peers, have been conducted. Conson et al. (2016) found that, in a hand laterality judgment task when presenting hand photographs of the backs and palms in four different orientations (i.e., 0, 90, 180, and 270°), individuals with ASD [18 participants (1 woman); mean (± SD) age = 14.6 ± 4.2; age range = 10–20] showed significantly longer RTs in comparison with TD peers, although Conson et al. (2013) showed that the RTs of individuals with Asperger syndrome [24 participants (3 women); mean (± SD) age = 13.4 ± 1.3; age range = 12–16] did not significantly differ from those of TD peers in the task using line-drawn 2D hand images. Furthermore, Chen et al. (2018) found that individuals with ASD [22 participants (2 women); mean (± SD) age = 13.47 ± 1.24; age range = 11–15] demonstrated significantly longer RTs in comparison with TD peers when performing the hand laterality judgment task while manipulating the angle combination (of 3D hand-arm image) within the frontal, sagittal, and transverse planes. The ratio of men in the samples of each of the above-mentioned ASD studies is greater than 87.5%, so the results of these studies reflect men’s task properties in hand laterality judgment. Of course, we must be cautious of the qualitative difference between an ASD diagnosis itself and a higher AQ score over the cutoff value; the current results, showing that longer RTs align with higher AQ scores in the men’s group, are not inconsistent with the results of these ASD studies.

Furthermore, the present study revealed in the laterality judgment task that the difference in RTs between the self and other conditions (other – self) are related to the AQ score only in the women’s group, indicating that women participants with (relatively) lower AQ scores react faster to a self-image than to the other’s image in the laterality judgment task, while women participants with (relatively) higher AQ scores show a reverse pattern. This result implies that, even in the present experiment with only two orientation conditions, and where it is assumed that a high sensorimotor load is not necessary for performance, the self-advantage could emerge in women who have (relatively) lower AQ scores. According to the “absent-self” hypothesis in ASD (Frith and Happé, 1999; Baron-Cohen, 2005; Frith and de Vignemont, 2005; Lombardo and Baron-Cohen, 2010; Lombardo et al., 2010), atypical self-awareness (for example, reduced distinction between the self and others) was observed in ASD. Such tendencies might (at least, partially) support the reverse pattern (i.e., self-disadvantage) in the women participants who show higher AQ scores. Why only the women’s group exhibited this relationship between the extent of self-(dis)advantage and autistic traits remains unclear. It could be influenced by the difference of the AQ distribution between men and women (for nonclinical populations; Ruzich et al., 2015), but this is unproven. Therefore, how visuospatial and motor abilities in each sex are modulated by autistic traits will need to be clarified in future studies.

Concerning the relationship to the BAS-2 score, an increase in dprime according to a higher BAS-2 score was found in the women’s group, but not in the men’s group, when performing the self-other discrimination task. This indicates that women who have greater body appreciation (i.e., higher BAS-2 scores) could better discriminate between themselves and others, suggesting that, for women, a more positive attitude toward their own body would lead to a better sensitivity to the body parts (in this case, the hands) when explicitly discriminating a self-image from an other’s image. Although the BAS-2 is applicable to both men and women, the significant relationship between RTs and BAS-2 scores only emerged in the women’s group. This implies that BAS-2 scores for each sex could reflect different contents. Indeed, BAS-2 scores could be related to eating disorder symptomatology for women, while the incremental variance in eating disorder symptomatology by BAS-2 scores did not reach significance for men (Tylka and Wood-Barcalow, 2015). In the future, these visual hand recognition tasks must be investigated in persons suffering from eating disorders. In fact, Campione et al. (2017) have already applied these visual hand tasks to eating disorder outpatient, but they did not report the results of the relationship between task performance and body image in the self-other discrimination task.

In summary, the men’s group showed a significant positive correlation between AQ scores and RTs in the laterality judgment task, while the women’s group showed a significant negative correlation between AQ scores and differences in RTs and a significant positive correlation between BAS-2 scores and dprime in the self-other discrimination task. These results suggest that men and women differentially adopt specific strategies (visual or motor simulation) and/or execution processes for implicit and explicit self-other discrimination of the hand, according to the different influences of autistic traits and the body appreciation on the visual recognition of body parts. The relatively small sample size of the current study is a limitation, therefore, a larger sample size is needed to clarify the detailed properties of hand recognition in a future study. The present finding of implicit and explicit self-other discrimination modulated by individual traits shaped by their cognitive and sensory-motor abilities provide deeper insight into how the self is shaped over the life-time.

## Data Availability Statement

The raw data supporting the conclusions of this article will be made available by the authors, without undue reservation.

## Ethics Statement

The studies involving human participants were reviewed and approved by Tokyo Metropolitan University Hino Campus. The patients/participants provided their written informed consent to participate in this study.

## Author Contributions

MK and TF conceived and designed the experiments, analyzed the data, and wrote the manuscript. MK performed the experiment. TF contributed reagents, materials and analysis tools. All authors contributed to the article and approved the submitted version.

### Conflict of Interest

The authors declare that the research was conducted in the absence of any commercial or financial relationships that could be construed as a potential conflict of interest.

## References

[ref1] Aranda C. Ruz M. Tudela P. Sanabria D. (2010). Focusing on the bodily self: the influence of endogenous attention on visual body processing. Atten. Percept. Psychophysiol. 72, 1756–1764. 10.3758/APP.72.7.1756, PMID: 20952775

[ref2] Avalos L. Tylka T. L. Wood-Barcalow N. (2005). The Body Appreciation Scale: development and psychometric evaluation. Body Image 2, 285–297. 10.1016/j.bodyim.2005.06.002, PMID: 18089195

[ref3] Baird G. Simonoff E. Pickles A. Chandler S. Loucas T. Meldrum D.. (2006). Prevalence of disorders of the autism spectrum in a population cohort of children in South Thames: the Special Needs and Autism Project (SNAP). Lancet 368, 210–215. 10.1016/S0140-6736(06)69041-7, PMID: 16844490

[ref4] Baron-Cohen S. (2005). “Autism—‘autos’: literally, a total focus on the self?” in The lost self: Pathologies of the brain and identity. eds. Feinberg T. E. Keenan J. P. (New York, NY: Oxford University Press), 166–180.

[ref5] Baron-Cohen S. Wheelwright S. Skinner R. Martin J. Clubley E. (2001). The autism-spectrum quotient (AQ): evidence from Asperger syndrome/high-functioning autism, males and females, scientists and mathematicians. J. Autism Dev. Disord. 31, 5–17. 10.1023/A:1005653411471, PMID: 11439754

[ref6] Bläsing B. Brugger P. Weigelt M. Schack T. (2013). Does thumb posture influence the mental rotation of hands? Neurosci. Lett. 534, 139–144. 10.1016/j.neulet.2012.11.034, PMID: 23201629

[ref7] Bracci S. Ietswaart M. Peelen M. V. Cavina-Pratesi C. (2010). Dissociable neural responses to hands and non-hand body parts in human left extrastriate visual cortex. J. Neurophysiol. 103, 3389–3397. 10.1152/jn.00215.2010, PMID: 20393066 PMC2888254

[ref8] Brady N. Maguinness C. Ní Choisdealbha Á. (2011). My hand or yours? Markedly different sensitivity to egocentric and allocentric views in the hand laterality task. PLoS One 6:e23316. 10.1371/journal.pone.0023316, PMID: 21826247 PMC3149647

[ref9] Campione G. C. Mansi G. Fumagalli A. Fumagalli B. Sottocornola S. Molteni M.. (2017). Motor-based bodily self is selectively impaired in eating disorders. PLoS One 12:e0187342. 10.1371/journal.pone.0187342, PMID: 29091967 PMC5665544

[ref10] Chan A. W. -Y. Peelen M. V. Downing P. E. (2004). The effect of viewpoint on body representation in the extrastriate body area. Neuroreport 15, 2407–2410. 10.1097/00001756-200410250-00021, PMID: 15640765

[ref11] Chen Y. -T. Tsou K. -S. Chen H. -L. Wong C. -C. Fan Y. -T. Wu C. -T. (2018). Functional but inefficient kinesthetic motor imagery in adolescents with autism spectrum disorder. J. Autism Dev. Disord. 48, 784–795. 10.1007/s10803-017-3367-y, PMID: 29119522

[ref12] Collins D. W. Kimura D. (1997). A large sex difference on a two-dimensional mental rotation task. Behav. Neurosci. 111, 845–849. 10.1037//0735-7044.111.4.845, PMID: 9267662

[ref13] Conson M. Aromino A. R. Trojano L. (2010). Whose hand is this? Handedness and visual perspective modulate self/other discrimination. Exp. Brain Res. 206, 449–453. 10.1007/s00221-010-2418-9, PMID: 20862462

[ref14] Conson M. De Bellis F. Baiano C. Zappullo I. Raimo G. Finelli C.. (2020). Sex differences in implicit motor imagery: evidence from the hand laterality task. Acta Psychol. 203:103010. 10.1016/j.actpsy.2020.103010, PMID: 31981826

[ref15] Conson M. Errico D. Mazzarella E. De Bellis F. Grossi D. Trojano L. (2015). Impact of body posture on laterality judgement and explicit recognition tasks performed on self and others’ hands. Exp. Brain Res. 233, 1331–1338. 10.1007/s00221-015-4210-3, PMID: 25633320

[ref16] Conson M. Hamilton A. De Bellis F. Errico D. Improta I. Mazzarella E.. (2016). Body constraints on motor simulation in autism spectrum disorders. J. Autism Dev. Disord. 46, 1051–1060. 10.1007/s10803-015-2652-x, PMID: 26572656

[ref17] Conson M. Mazzarella E. Frolli A. Esposito D. Marino N. Trojano L.. (2013). Motor imagery in Asperger syndrome: testing action simulation by the hand laterality task. PLoS One 8:e70734. 10.1371/journal.pone.0070734, PMID: 23894683 PMC3720915

[ref18] Conson M. Volpicella F. De Bellis F. Orefice A. Trojano L. (2017). “Like the palm of my hands”: motor imagery enhances implicit and explicit visual recognition of one’s own hands. Acta Psychol. 180, 98–104. 10.1016/j.actpsy.2017.09.006, PMID: 28926731

[ref19] Cooper L. A. Shepard R. N. (1975). Mental transformations in the identification of left and right hands. J. Exp. Psychol. Hum. Percept. Perform. 104, 48–56. PMID: 1141835

[ref20] De Bellis F. Trojano L. Errico D. Grossi D. Conson M. (2017). Whose hand is this? Differential responses of right and left extrastriate body areas to visual images of self and others’ hands. Cogn. Affect. Behav. Neurosci. 17, 826–837. 10.3758/s13415-017-0514-z, PMID: 28536919

[ref21] de Lange F. P. Helmich R. C. Toni I. (2006). Posture influences motor imagery: an fMRI study. NeuroImage 33, 609–617. 10.1016/j.neuroimage.2006.07.017, PMID: 16959501

[ref22] Downing P. E. Jiang Y. Shuman M. Kanwisher N. (2001). A cortical area selective for visual processing of the human body. Science 293, 2470–2473. 10.1126/science.1063414, PMID: 11577239

[ref23] Ferri F. Frassinetti F. Ardizzi M. Costantini M. Gallese V. (2012). A sensorimotor network for the bodily self. J. Cogn. Neurosci. 24, 1584–1595. 10.1162/jocn_a_00230, PMID: 22452562

[ref24] Ferri F. Frassinetti F. Costantini M. Gallese V. (2011). Motor simulation and the bodily self. PLoS One 6:e17927. 10.1371/journal.pone.0017927, PMID: 21464959 PMC3064658

[ref25] Frassinetti F. Ferri F. Maini M. Benassi M. G. Gallese V. (2011). Bodily self: an implicit knowledge of what is explicitly unknown. Exp. Brain Res. 212, 153–160. 10.1007/s00221-011-2708-x, PMID: 21553263

[ref26] Frassinetti F. Maini M. Romualdi S. Galante E. Avanzi S. (2008). Is it mine? Hemispheric asymmetries in corporeal self-recognition. J. Cogn. Neurosci. 20, 1507–1516. 10.1162/jocn.2008.20067, PMID: 18211238

[ref27] Frassinetti F. Pavani F. Zamagni E. Fusaroli G. Vescovi M. Benassi M.. (2009). Visual processing of moving and static self body-parts. Neuropsychologia 47, 1988–1993. 10.1016/j.neuropsychologia.2009.03.012, PMID: 19428432

[ref28] Frith U. de Vignemont F. (2005). Egocentrism, allocentrism, and Asperger syndrome. Conscious. Cogn. 14, 719–738. 10.1016/j.concog.2005.04.006, PMID: 15996486

[ref29] Frith U. Happé F. (1999). Theory of mind and self-consciousness: what is it like to be autistic? Mind Lang. 14, 82–89. 10.1111/1468-0017.00100

[ref30] Gentilucci M. Daprati E. Gangitano M. (1998). Implicit visual analysis in handedness recognition. Conscious. Cogn. 7, 478–493. 10.1006/ccog.1998.0368, PMID: 9787057

[ref31] Hannay H. J. Ciaccia P. J. Kerr J. W. Barrett D. (1990). Self-report of right-left confusion in college men and women. Percept. Mot. Skills 70, 451–457. 10.2466/pms.1990.70.2.451, PMID: 2342844

[ref32] Ionta S. Blanke O. (2009). Differential influence of hands posture on mental rotation of hands and feet in left and right handers. Exp. Brain Res. 195, 207–217. 10.1007/s00221-009-1770-0, PMID: 19326106

[ref33] Ionta S. Fourkas A. D. Fiorio M. Aglioti S. M. (2007). The influence of hands posture on mental rotation of hands and feet. Exp. Brain Res. 183, 1–7. 10.1007/s00221-007-1020-2, PMID: 17643238

[ref34] Linn M. C. Petersen A. C. (1985). Emergence and characterization of sex differences in spatial ability: a meta-analysis. Child Dev. 56, 1479–1498. 10.2307/1130467, PMID: 4075870

[ref35] Lombardo M. V. Baron-Cohen S. (2010). Unraveling the paradox of the autistic self. Wiley Interdiscip. Rev. Cogn. Sci. 1, 393–403. 10.1002/wcs.45, PMID: 26271379

[ref36] Lombardo M. V. Chakrabarti B. Bullmore E. T. Sadek S. A. Pasco G. Wheelwright S. J.. (2010). Atypical neural self-representation in autism. Brain 133, 611–624. 10.1093/brain/awp306, PMID: 20008375

[ref37] Lust J. M. Geuze R. H. Wijers A. A. Wilson P. H. (2006). An EEG study of mental rotation-related negativity in children with developmental coordination disorder. Child Care Health Dev. 32, 649–663. 10.1111/j.1365-2214.2006.00683.x, PMID: 17018041

[ref38] Macmillan N. A. Creelman C. D. (1991). Detection theory: A user’s guide. New York, NY: Cambridge University Press.

[ref39] Mandy W. Charman T. Gilmour J. Skuse D. (2011). Toward specifying pervasive developmental disorder-not otherwise specified. Autism Res. 4, 121–131. 10.1002/aur.178, PMID: 21298812

[ref40] Mochizuki H. Takeda K. Sato Y. Nagashima I. Harada Y. Shimoda N. (2019). Response time differences between men and women during hand mental rotation. PLoS One 14:e0220414. 10.1371/journal.pone.0220414, PMID: 31348807 PMC6660072

[ref41] Namatame H. Uno K. Sawamiya Y. (2017). Development of Japanese version of the Body Appreciation Scale-2. Shinrigaku Kenkyu 88, 358–365. 10.4992/jjpsy.88.16216

[ref42] Ní Choisdealbha Á. Brady N. Maguinness C. (2011). Differing roles for the dominant and non-dominant hands in the hand laterality task. Exp. Brain Res. 211, 73–85. 10.1007/s00221-011-2652-9, PMID: 21533699

[ref43] Oldfield R. C. (1971). The assessment and analysis of handedness: the Edinburgh inventory. Neuropsychologia 9, 97–113. 10.1016/0028-3932(71)90067-4, PMID: 5146491

[ref44] Parsons L. M. (1987). Imagined spatial transformations of one’s hands and feet. Cogn. Psychol. 19, 178–241. 10.1016/0010-0285(87)90011-9, PMID: 3581757

[ref45] Parsons L. M. (1994). Temporal and kinematic properties of motor behavior reflected in mentally simulated action. J. Exp. Psychol. Hum. Percept. Perform. 20, 709–730. 10.1037//0096-1523.20.4.709, PMID: 8083630

[ref46] Parsons L. M. Gabrieli J. D. Phelps E. A. Gazzaniga M. S. (1998). Cerebrally lateralized mental representations of hand shape and movement. J. Neurosci. 18, 6539–6548. 10.1523/JNEUROSCI.18-16-06539.1998, PMID: 9698341 PMC6793195

[ref47] Peelen M. V. Downing P. E. (2005). Is the extrastriate body area involved in motor actions? Nat. Neurosci. 8:125. 10.1038/nn0205-125a, PMID: 15682180

[ref48] Peelen M. V. Downing P. E. (2007). The neural basis of visual body perception. Nat. Rev. Neurosci. 8, 636–648. 10.1038/nrn2195, PMID: 17643089

[ref49] Reijonen J. H. Pratt H. D. Patel D. R. Greydanus D. E. (2016). Eating disorders in the adolescent population: an overview. J. Adolesc. Res. 18, 209–222. 10.1177/0743558403018003002

[ref50] Rossetti Y. Holmes N. Rode G. Farnè A. (2010). “Cognitive and bodily selves: how do they interact following brain lesion?” in The embodied self: Dimensions, coherence and disorders. eds. Fuchs T. Sattel H. Henningsen P. (Stuttgart: Schattauer Verlag), 117–133.

[ref51] Ruzich E. Allison C. Smith P. Watson P. Auyeung B. Ring H.. (2015). Measuring autistic traits in the general population: a systematic review of the Autism-Spectrum Quotient (AQ) in a nonclinical population sample of 6,900 typical adult males and females. Mol. Autism 6:2. 10.1186/2040-2392-6-2, PMID: 25874074 PMC4396128

[ref52] Saxe R. Jamal N. Powell L. (2006). My body or yours? The effect of visual perspective on cortical body representations. Cereb. Cortex 16, 178–182. 10.1093/cercor/bhi095, PMID: 15858162

[ref53] Sekiyama K. (1982). Kinesthetic aspects of mental representations in the identification of left and right hands. Percept. Psychophys. 32, 89–95. 10.3758/BF03204268, PMID: 7145594

[ref54] Shepard R. N. Metzler J. (1971). Mental rotation of three-dimensional objects. Science 171, 701–703. 10.1126/science.171.3972.701, PMID: 5540314

[ref55] Steenbergen B. van Nimwegen M. Crajé C. (2007). Solving a mental rotation task in congenital hemiparesis: motor imagery versus visual imagery. Neuropsychologia 45, 3324–3328. 10.1016/j.neuropsychologia.2007.07.002, PMID: 17706255

[ref56] Striegel-Moore R. H. Bulik C. M. (2007). Risk factors for eating disorders. Am. Psychol. 62, 181–198. 10.1037/0003-066X.62.3.181 17469897

[ref57] ter Horst A. C. van Lier R. Steenbergen B. (2010). Mental rotation task of hands: differential influence number of rotational axes. Exp. Brain Res. 203, 347–354. 10.1007/s00221-010-2235-1, PMID: 20376435 PMC2871105

[ref58] Tylka T. L. Wood-Barcalow N. L. (2015). The Body Appreciation Scale-2: item refinement and psychometric evaluation. Body Image 12, 53–67. 10.1016/j.bodyim.2014.09.006, PMID: 25462882

[ref59] Voyer D. Voyer S. Bryden M. P. (1995). Magnitude of sex differences in spatial abilities: a meta-analysis and consideration of critical variables. Psychol. Bull. 117, 250–270. 10.1037/0033-2909.117.2.250, PMID: 7724690

[ref60] Wakabayashi A. Tojo Y. Baron-Cohen S. Wheelwright S. (2004). The Autism-Spectrum Quotient (AQ) Japanese version: evidence from high-functioning clinical group and normal adults. Shinrigaku Kenkyu 75, 78–84. 10.4992/jjpsy.75.78, PMID: 15724518

[ref61] Zapparoli L. Invernizzi P. Gandola M. Berlingeri M. De Santis A. Zerbi A.. (2014). Like the back of the (right) hand? A new fMRI look on the hand laterality task. Exp. Brain Res. 232, 3873–3895. 10.1007/s00221-014-4065-z, PMID: 25150553

